# Quantity Statistics and Internal Control

**Published:** 1924-12

**Authors:** J. E. Stone

**Affiliations:** Incorporated Accountant


### QUANTITY STATISTICS AND INTERNAL CONTROL.

By J. E. STONE, F.S.A.A., F.S.S., F.R.Econ.S., Incorporated Accountant.

In any well managed hospital, and particularly in one of large scale, there must necessarily be a very great dependence on statistics. Efficient and economical administration implies the possession and recognition of facts and acting in the light of their interpretation. From this it follows that genuine statistical control must begin with the desire of the Executive to base their decisions so far as practicable on a careful analysis of the facts which relate to the hospital. Assuming that a genuine desire exists on the part of the Executive for statistics as an element of control, the first question is the form they are to take. Are they to be statistics of monetary values or of quantities ?

### Quantities and Prices.

Instances will no doubt occur to the reader of cases where statistics of quantities are absolutely essential, as, for example, when any change takes place in the purchasing power of money. In times of fluctuating prices comparisons of monetary values alone do not afford sufficient information upon which to form conclusions and base judgments. If the price of a commodity is reduced and the quantity consumed measured in terms of value remains the same, it is obvious that an increase in consumption has taken place. This fact, however, would not be disclosed by the financial statistics, and it is quite possible for the financial statistics to show a reduction in cost in spite of consumption having increased.

### Detecting Excessive Consumption.

The main object in the preparation of statistics of quantities is to locate excessive consumption, and for this object to be accomplished it is necessary that the figures shall be prepared in such detail and presented in such form that the consumption in each ward and department may be easily comprehended and compared. Statistics of total expenditure and consumption are useless. For instance, no control can be exercised over the cost and consumption of provisions if only total cost and total quantity statistics are prepared. The figures must show the cost and consumption in respect of each class receiving meals. Further, it will readily be conceived that the actual quantities consumed in the various wards cannot be used as a basis for comparison, owing to the differences in numbers treated. The consumption figures have, therefore, to be reduced to a common unit in order to enable their real meaning to be more readily grasped and changes more easily detected and valued. This unit, which may be the per occupied bed or the per 100 patient days, is adopted solely for the purpose of reducing the figures to a common basis to allow of just comparisons being made.

### The Question of Relativity.

In making these comparisons it should be remembered that facts, statistical or otherwise, are always relative, and the statistical data which expresses them, although definite, are not to be interpreted in an absolute sense. The measurements are relative, and they must be considered in connection with the problems and the conditions to which they apply. One of the chief difficulties experienced in making comparisons over wards is the chance of fundamental differences in the class of patients treated, and the variation in admissions.

### Quantity Statistics.

The preparation of trustworthy quantity statistics presupposes the existence of a system of accounting for stores and of rigidly observed regulations regarding issues. In this connection it may be pointed out that for every duty imposed upon a cashier with regard to cash, there should be imposed upon the storekeeper a corresponding duty with regard to stores. Efficient buying may save anything from 10 to 15 per cent., but an efficient system of stores control may save an untold percentage. Moreover, cases are not unknown where the saving made by economical buying is more than counterbalanced by the absence of any method of stores control.

### Combining Values and Quantities.

Quantity statistics will not provide all the information necessary for drawing correct conclusions. Statistics of monetary values are of no less importance than those relating to quantities, and it is a question for consideration whether the best results are not obtained by a judicious combination of the two methods. It is only possible in a short article of this kind to touch upon the fringe of the subject, and many points have had to be left out which are of importance. Among these points may be mentioned :— 1. Methods of collecting the necessary statistics and the form of statistical tables to be employed. 2. Average consumption and cost figures, and what they really mean. 3. Factors to be considered when making comparisons. 4. Frequency of reports, and their circulation to interested parties. 5. Use of graphical charts to represent statistical data (these are of the greatest value provided they are charts of vital figures, and not totals). 6. Storage of statistical reports.

### A Bureau of Hospital Statistics.

I am strongly in favour of the establishment of a Bureau of Hospital Statistics. At present each hospital is a separate unit struggling along in the darkness and fighting its own battles more or less in ignorance of the doings of similar hospitals. The result is that each has to learn the methods of economical administration in the school of experience —an efficient but admittedly an expensive school. The establishment of such a Bureau would do much towards breaking down the barrier of isolation which exists at present, and bringing about closer co-operation. Internal and external statistics, including quantities, prices, costs of services, and many other items, would be available to hospital officers, and it would be possible to ascertain what other hospitals were doing, what they were paying for commodities, their consumption and cost statistics, rates of pay, schedules of duties, etc., and the various methods employed in different hospitals to secure an efficient and economical system of administration.

